# Supplementary material for: Interventions to Improve Treatment Outcomes among Adolescents on Antiretroviral Therapy with Unsuppressed Viral Loads: A Systematic Review
Source: Int J Environ Res Public Health. 2022 Mar 25;19(7):3940. doi: 10.3390/ijerph19073940 (PMC8997420; doi:10.3390/ijerph19073940)
Supplement: Supplementary file 1 [file ijerph-19-03940-s001.zip › ijerph-1623032-supplementary.pdf]

Table S1: Systematic Review Search Strategy

Search criteria based on PICOT elements	Population: Adolescents aged 10-19 years living with HIV, acquired perinatally or behaviourally<br>Intervention: Any group-based or individual-level interventions to improve treatment outcomes targeting adolescents who have challenges suppressing their viral loads, including Differentiated Service Delivery (DSD) models (at facility, community or homebased levels).<br>Comparison: Studies reporting comparisons on quantitative measures of retention, adherence and viral load suppression between intervention and standard care (pre-/post- or multi-arm comparison groups).<br>Outcome: Primary outcome – viral load suppression, Secondary outcomes – retention in care and ART adherence<br>Time: 2010 – 2020
Search strategy used for PubMed	(adolescents or teenagers or young adults or teen or youth or student or adolescence) AND (antiretroviral therapy) AND (retention) AND (adherence or compliance) AND (viral load) AND (suppression) AND (hiv or aids or acquired human immunodeficiency syndrome or human immunodeficiency virus)
Search strategy used for Sabinet	[[All: adolescents] OR [All: teenagers] OR [All: young]] AND [[All: adults] OR [All: teen] OR [All: youth] OR [All: student]] AND [All: or adolescence] AND [All: antiretroviral] AND [[[All: therapy] AND [All: retention] AND [All: adherence]] OR [[All: compliance] AND [All: viral]]] AND [All: load] AND [All: suppression]
Search strategy used for EBSCOhost	"adolescents or teenagers or young adults or teen or youth AND antiretroviral therapy or antiretroviral treatment AND adherence AND retention AND viral load AND HIV Published Date: 20100101-20201231 AND Apply equivalent subjects on 2021-03-15 10:19 AM", Limiters - Published Date: 20100101-20201231, Expanders - Apply equivalent subjects, Narrow by Language: - English, Narrow by Subject Age: - child: 6-12 years, Narrow by Subject Age: - adolescent: 13-18 years, Search modes - SmartText Searchin
Search strategy used for ScienceDirect	adolescents AND antiretroviral therapy AND retention AND adherence OR compliance AND viral load AND suppression AND hiv or aids
Search strategy used for Scopus	TITLE-ABS-KEY ((adolescents OR teenagers OR young AND adults OR teen OR youth OR student OR adolescence) AND (antiretroviral AND therapy) AND (retention) AND (adherence OR compliance) AND (viral AND load) AND (suppression) AND (hiv OR aids OR acquired AND human AND immunodeficiency AND syndrome OR human AND immunodeficiency AND virus))
Search strategy used for Web of Science	((adolescents or teenagers or young adults or teen or youth or student or adolescence) AND (antiretroviral therapy) AND (retention) AND (adherence or compliance) AND (viral load) AND (suppression) AND (hiv or aids or acquired human immunodeficiency syndrome or human immunodeficiency virus))<br>Timespan: 2010-2020. Indexes: SCI-EXPANDED, SSCI, A&HCI, CPCI-S, CPCI-SSH, BKCI-S, BKCI-SSH, ESCI.
Limiters	Age: 10-19 years, Languages: English, Publication Date: (01/01/2010 TO 12/31/2020),

Table S2: Data extraction matrix

**Reviewer: FKM**					
**GENERAL**					
**Study ID** *(Surname) [Year]*	Nasuuma *et al*, [2018]	Mavhu *et al*, [2020]	Ssewamala *et al*, [2020]	Ndhlovu *et al*, [2021]	Ekwunife et al, [Pre-print]
**Country/countries**	Uganda	Zimbabwe	Uganda	Zimbabwe	Nigeria
**Study Design**	**Retrospective Cohort**, chart review	**Cluster RCT**, thematic analysis of in-depth qualitative data	5-year longitudinal **Cluster RCT**	**RCT**	**Cluster RCT**
**STUDY POPULATION**					

**Source (s) of study poplation**	15 Public health facilities, Urban + Rural	16 PHC clinics, 2 rural districts with lowest ART coverage, 29%	39 Healthcare clinics, 5 districts	1 Referral Hospital, Parirenyatwa Hospital Family Care Clinic	12 Hospitals registered with National Agency for the Control of AIDS
**Inclusion criteria**	All ART-treated children 9M-19Y with VL>1000	ALHIV, on ART at selected sites, 13-19 yrs, starting or already on ART, able to assent/ consent from caregiver if <18 yrs	Clinic providing adolescent HIV services, Adolescents 10-16, Disclosed HIV+ status, on ART, registered at one of the clinics, non-institutionalized, detectable VL	10 - 24 years, with virologic failure defined as >400 copies/ml, 2 consecutive >1 Month apart, or >400 within last 12 months	Adolescents 10-19 years, initiated into HIV care and antiretroviral therapy for minimum of 6 months, off ART despite attempts to reengage, with detectable VL>20 copies/ml
**Exclusion criteria**	Did not attend 3 IAC sessions	Physically unwell to attend clinic (bedridden), psychotic, unable to give informed assent or consent	Undisclosed HIV status, under or over age, unable to comprehend study procedures	Adolescents who could not provide informed assent or consent, Adolescents previously or currently exposed to Zvandiri	
**Recruitment procedure** *(including any randomization)*	All ART-treated children 9M-19Y with VL>1000	Public clinics were units of randomization, adolescents lists created from pre-ART and ART registers. Randomization by random number tables generated in Stata by an independent statistician	Adolescents were screened at the selected randomized sites for eligibility and, llocation to groups done based on clusters to avoid contamination or cross-overs (two-arm, cluster randomization design)	PHFCC + 4 other facilities in Harare, screened patients for enrolment, Randomization by computer generated randomization scheme. Allocation concealed in opaque envelopes.	Hospitals paired into units (tertiary, secondary), radomly selected to intervention or control group, 6 in each group, randomisation using Research Randomiser, a web-based computer random-number generator
**Population characteristics** *(age, sex, medical background, epidemiological characteristics)*	9M - 19 years with VL>1000	13 - 19 years, 47% had VL>1000	10 - 16 years	10 - 24 years, with virologic failure defined as >400 copies/ml, 2 consecutive >1 Moth apart, or >400 within last 12 months	Adolescents 10-19 years, off ART despite attempts to reengage, with detectable VL>20 copies/ml
**Number recruited**	N=192	N=496	N=702	N=212	N=246
**Number included/analyzed** *(sample size)*	N=192	N=479 analyzed	288 adolescents with detectable VL at baseline	N=134 (63.2%) were adolescents 10-19 years	N=246
**DESCRIPTION OF INTERVENTION**					
**Intervention type**	**Intensified Adherence Counseling (IAC)** sessions conducted for children and adolescents with VL≥1000 copies/ml. Also	**Peer-led multicomponent DSD intervention**. Intervention group (enhanced HIV care support) were allocated a Community	**Family based economic empowerment intervention**. In addition to medical (ART) and psychosocial (Adherence) SOC, intervention group	**Community-based peer support intervention** to improve virologic suppression. SOC Primary Care Couselors do individual and group sessions. Intervention group	**Conditional Economic Incentives and Motivational Interviewing** to Improve viral suppression, adherence to ART and retention in Care. The study

	involves trained expert patients, uses the 5 As (Assess, Advise, Assist, Agree, Arrange). A repeat VL done after 3 monthly sessions with good adherence	Adolescent Treatment Supporter (CATS), attended monthly support group, received texts, calls, home visits & clinic-based counseling, Vs standard HIV care. HIV vulnerability assessed every 3 months, caregivers invited to a support group. All clinics received adherence counseling.	received child development accounts (incentivised savings accounts) and microenterprise workshops, for medical expenses and education related expenses.	enrolled in Zvandiri program.	primarily looked at cost-effectiveness and feasibility of the incentive scheme
**Follow-up period/duration**	June 2015- Dec 2016 (19 months)	August 2016-March 2017 (8 months enrolment), 96 weeks endline	5 years	July 2016- February 2018, (24 and 36 weeks)	2 years (intervention and post-intervention years)
**OUTCOME DEFINITION**					
**Viral Suppression**	VL<1000 copies/ml	VL<1000 copies/ml Repeat VLs midline (42-60 weeks) after 96 weeks	VL<40 copies/ml Month 0, 12, 24, 36, 48 post intervention	VL<1000 copies/ml Repeat VL at 12, 24 and 36 weeks	VL<20 copies/ml Month 12 and 24
**Adherence to ART**		Attending <80% of scheduled visits		Self-reported adherence, periodic pill counts	
**Retention in HIV Care**		WHO definition of RiC			
**Others**	ART regimen switch	Death, Cost-effectiveness, Depression, QoL		Baseline Drug Resistance mutations (DRMs) on those with VL≥1000, CD4	Cost-effectiveness
**OUTCOME MEASURES**					
**Viral Suppression**	Proportions and Percentages	Prevalence Ratio	Incidence Rate Ratio	Odd Ratio	Percentage Difference in VLS
**Adherence to ART**				Percentage (%)	
**Retention in HIV Care**		Proportion not retained			
**RESULTS**					
**Viral Suppression**	Overall viral suppression (10 – 19 years): **29%**<br><br>10-14yrs: 51 (25.6%) unsuppressed, 26	Risk Ratio= **1.17** (95% CI 1.04 – 1.32)<br><br>Adjusted Prevalence Ratio= 0.58 (95% CI	Incidence Rate Ratio= **1.468** (95% CI 1.064 - 2.038, *p=0.008*).<br><br>Intervention incidence of	Adjusted OR = **1.14** (95% CI 0.82 - 1.59), p=0.439. At 24 weeks:<br><br>Intervention group detectable viraemia	Overall difference of **11.7%** suppression<br>Completed trial is currently under peer review for publication.

	(41.3%) suppressed. 15-19yrs: 32 (16.1%) unsuppresed, 8 (12.7%) suppressed, *p=0.175*	0.36 - 0.94, *p=0.03*). Intervention (Zvandiri) =52/209 (25%) had VL≥1000, Control =97/270 (36%) had VL≥1000	undetected VL = 0.254, Control =0.173 158 intervention group (20 clinics), 130 in non-intervention group (19 clinics)	=79 (76%), Control =96 (89%), p=0.013. At 36 weeks: 71 (68.3%) vs 86 (79.6%). Overall suppression rates poor at 31.7% Intervention vs 20.4% in SOC, with high levels of NNRTI DRMs in both arms at baseline. 86.3% had VL≥1000 at baseline	At baseline: Intervention 21.8% (26/119) with undetected VL, at 12 months 31.9% (38/119), 10.1% difference. Control 42.5% (54/127), 40.9% (52/127), -1.6% diff. Adjusted with Delutogravir 14.6% vs 5.7%.
**Adherence to ART**				66.0%-67.8% adherence in the intervention group and 68.9%-81.1% in the standard of care group, from baseline to week 36	
**Retention in HIV Care**		Adjusted prevalence ratio of discontinuation of ART for ≥3 months at 0.68 (95% CI 0.23 – 1.99, p = 0.45) and adjusted prevalence ratio of attendance <80% of scheduled visits at 0.80 (95% CI 0.32 – 2.02, p = 0.62)			
**Others**		Annual cost of$450.36 in standard of care clinics, Vs $1340.00 for adolescents in the Zvandiri intervention			$170.30 in routine care Vs intervention cost of $356.70 per adolescent per annum
**RISK OF BIAS ASSESSMENT** (ROBINS-I or CASP)	ROBINS-I	CASP	CASP	CASP	CASP
**Bias due to confounding**	Low				
**Bias in selection of participants into the study**	Low				
**Bias in classification of interventions**	Low				
**Bias due to deviations from intended interventions**	Moderate				
**Bias due to missing data**	Moderate				
**Bias in measurement of outcomes**	Low				
**Bias in selection of the reported result**	Low				
**CASP Section A: Is the basic study design valid for a RCT?**					

**1.Did the study address a clearly focused research question?**		Yes	Yes	Yes	Yes
**2.Was the assignment of participants to interventions randomised?**		Yes	Yes	Yes	Yes
**3.Were all participants who entered the study accounted for at its conclusion?**		(modified ITT)	(per-protocol analysis)	(study terminated due to funding)	Can`t tell
**Section B: Was the study methodologically sound?**					
**4.Were participants, investigators, assessors/analysers "blinded"?**		No (not possible)	No (not possible)	No (not possible)	No (not possible)
**5.Were the study groups similar at the start of the RCT?**		Yes	Yes	Yes	Yes
**6.Apart from experimental intervention, did each group receive same level care?**		Yes	Yes	Yes	No (Intervention group had more VL tests)
**Section C: What are the results?**					
**7.Were the effects of intervention reported comprehensively?**		Yes	Can`t tell	Yes	Can`t tell
**8.Was the precision of the estimate of the intervention/treatment effect reported?**		Yes	Yes	Yes	No
**9.Do the benefits of the experimental intervention outweigh the harms/costs?**		Yes	Yes	Yes	Yes
**Section D: Will the results help locally?**					
**10.Can the results be applied to your local population/in your context?**		Yes	Yes	Yes	Yes
**11.Would the experimental intervention provide greater value to people in your care?**		Yes	Yes	Yes	Yes
**Risk of bias judgement**	MODERATE				
**GRADE (certainty of evidence)**					
**Risk of Bias**	Moderate	Low	Low	Moderate	Moderate
**Consistency**					
**Directness**	High	High	High	High	Low
**Imprecision**		Low	Moderate	Moderate	
**Publication bias**					
**Final Quality of Evidence**	**Low**	**High**	**Moderate**	**Moderate**	**Low**
	Cohort study downgraded by 1 due to non-radomized study design		Downgraded by 1 due to imprecicion, wide 95% CI	Downgraded by 1 due to incomplete data, study not completed as planned, wide 95% CI	Downgraded by 2 due to high Risk of Bias, no CI reported, missing information
**Funding source**	No additional funding	Declared ViiV Healthcare	Declared NICHD	Declared PEPFAR	European Union (EDCTP2 Programme)
